# The Medical Society of the County of Erie

**Published:** 1909-06

**Authors:** Franklin C. Gram

**Affiliations:** Secretary


### SOCIETY PROCEEDINGS.

### The Medical Society of the County of Erie.

### Regular Quarterly Meeting, April 19, 1909.

REPORTED BY FRANKLIN C. GRAM, M. D., Secretary.

ONE of the largest audiences, as well as one representing every branch of the medical profession, gathered at the University Club, on the evening of Monday, April 19, 1909, when the president, Dr. Charles A. Wall, at 9 o'clock, called the regular meeting of the Medical Society of the County of Erie to order.

The minutes of the regular meeting of the society held February 19, 1909, as well as the minutes of the meetings of the council held March 1, 1909, and April 5, 1909, were read by the secretary and approved.

The approval of these minutes also included the adoption of the action to confer honorary life membership upon Dr. Ernest Wende.

The treasurer, Dr. Lytle, reported that Dr. Roy G. Strong, whose membership had been dropped, had again fulfilled all the requirements of the by-laws and was, therefore, entitled to reinstatement, and on motion, Dr. Roy G. Strong was reinstated to active membership.

Dr. Thomas H. McKee, Chairman of the Committee on Membership, reported favorably upon the following applications for membership in the society, and on motion, they were duly elected: Edward M. Dooley, 406 Louisiana street; Myrtle A. Hoag, 426 Dearborn street; Frank B. Rasbach, 172 Allen street; Caroline Lichtenberg, 24 Orange street; Valentine A. Decot, 1099 Genesee street; Max Breuer, 33 Allen street; Joseph S. Lewis, 258 Elmwood avenue; Clara Anna March, 465 Ashland avenue; Wm. H. Mansperger, 1003 Main street; M. Louise Mallory, 14 Villa avenue; Hyatt Regester, 1106 Main street, all of Buffalo, and F. K. Armstrong, Eden Centre, N. Y.

Dr. Julius Ullman, Chairman of the Auditing Committee, reported that examination of the treasurer's books for the year 1908 showed them to be correct.

Dr. John H. Grant, Chairman of the Board of Censors, reported as follows:

January 18—Case against Lambert, practising medicine without a license—personating another practitioner and having in his possession a fake certificate. Indicted by Grand Jury on three counts. Plead guilty in Supreme Court and sentenced to six months' imprisonment in Erie County Penitentiary. Since sent to asylum for Criminal Insane at Matteawan.

January 27—F. G. O. Ehle, posing as the Ebenezer Medicine Company, was arrested for doing business without being registered in the Erie County Clerk's Office, and for practising medicine without a license. Found guilty of first offense in Police Court and fined $5. Being a cripple in his hands, the acting Police Judge took sympathy on him; said the evidence in the practice of medicine was not conclusive and discharged Ehle with a warning.

January 27—One, J. W. Merrow, posing as the Atlantic Clinic, upstairs, No. 7 East Swan St., Buffalo, where he had set up elaborate offices under cover of one, Dr. Richard E. Hawken, a graduate of Toronto, and endorsed by New York Regents, and Dr. Winters, Detroit College of Medicine—1886, is now being investigated and to secure evidence toward having Hawken's and Winter's state license revoked for deceit in covering up the illegal practice of medicine. Merrow's case—a bad one—his license to practise in Maine was revoked for being falsely procured; also in Ohio, where he served a term in the county prison for fraudulent use of the mails; and he was fined and ordered out of the State; at Syracuse, for practising without authority. He claimed, in Maine, to be a graduate of a medical college in Indianapolis, long since defunct, and on inquiry by the Main Board, the college building had been burned down and the records destroyed. Measures will be soon taken to present this case to the Regents, as regards Drs. Hawken and Winters, and the use of the mails by Merrow.

Since writing the above, the Atlantic Clinic, including Merrow, Hawken and Winters, have, like the Arabs in the night, silently stolen away. They have gone into new pastures among the green hills of Vermont. I have just located them there.

February 6—The case of one, C. G. Edwards, posing as the "Nature's Creation Remedy Company," at 316 Franklin Street, and now of 531 Brisbane Building, has been under observation. Your chairman caused a sickly looking young man (but really quite healthy), in company with a witness, to present himself to Edwards, and the young man was told by Edwards that he had "consumption" and that he could cure him in a short time.

"Nature's Creation" has been partially analyzed and may be said to consist of vegetable matters, such as burdock root and other roots that grow by the wayside, and iodide of potash.

March 20th, a warrant was sworn out for Edwards, and he was arrainged in the Police Court, March 20. Case adjourned to March 23d, on which date he was held to the Grand Jury under $500 bond. The case has been heard recently before the Grand Jury and its verdict is awaited with some misgiving.

March 11—Recently the so-called "Vacuum Company" has been under investigation by the Post Office authorities, through the efforts of members of the board, and a fraud order against the concern was issued by the post office department: the wares advertised having been found wholly without efficacy in the cure of disease.

April—Strange to say, a man has been practising in Buffalo for 20 years without lawful registration—one, William Bell, of Cazenovia street. He has a diploma issued in 1889 by a Therapen-Electro College at Indianapolis, Ind. His case, at this writing, is before the Police Court. He has asked delay in the hope that the Regents may grant him an examination.

April 10—One, H. James Ince, who was convicted of performing abortions and sentenced in October, 1907, to one year in the penitentiary, has applied to be restored to practise under the medical law and a hearing was given before a committee of the State Board of Medical Examiners in this city on April 10, 1909. Your Board of Censors was represented at the hearing, and they strongly opposed this man's restoration to practise in New York, on the ground that his crimes covered a number of years (7 in all), and his period of probation was too short to determine the sincerity of his reformation.

This report of the Board of Censors was duly received, and the thanks of the society tendered to the board, and to Dr. Grant in particular, for the excellent showing.

After the regular business of the society had been disposed of, President Wall briefly stated that, at a previous meeting, the society had elected Dr. Henry Reed Hopkins an honorary life member in recognition of his forty years' service as a member of the Board of Censors. The society had likewise decided to make the presentation of this membership an event in its history. Various speakers had, therefore, been designated to accentuate some of Dr. Hopkins's many sided activities during his long professional career.

The first speaker of the evening was Dr. Charles G. Stockton, President of the Medical Society of the State of New York. Dr. Stockton said that while he was an interne in the Buffalo General Hospital, he had a little talk with Dr. Hopkins, who was then a visiting physician at that institution. Doubtless Dr. Hopkins had long forgotten the incident, but not so the speaker, who had profited by the friendly advice of a well trained mind given years ago. He referred to Dr. Hopkins's well known methods of doing things, which he compared to the flight of a meteor going through the skies regardless of the other heavenly bodies. If the doctor was satisfied as to the right or wrong of any question, it did not take him long to act. Policy nor personal consideration never entered the game. For this reason, there was probably no physician in Buffalo who had made more enemies. There was, likewise, no general practitioner in the state who was better known. He is an ex-president of the state society, and for years was one of its most active members. His activities regarding state medical legislation are matters of history. When a good medical bill required support, the good doctor was on hand, and when a vicious bill was up, the doctor was likewise at the fore, and he usually succeeded in convincing the committee by his sound, logical methods of reasoning. Dr. Stockton conveyed the greetings of the state society, and closed with personal expressions of sincere good will.

Dr. William Warren Potter, president of the New York State Board of Medical Examiners, gave a concise history of the medical legislation of the last quarter of a century which lead up to the present state licensing law. Coming from one who was intimately and continuously associated with this work from the beginning, his remarks were of exceeding interest. This law had its inception in the Medical Society of the County of Erie, and Dr. Hopkins was the originator and prime mover. Dr. Potter traced the various steps which the law underwent, the many different interests who opposed every move, the sometimes absurd concessions which had to be made by friends of the measures in order to get any kind of medical law on the statute books, the composition of the original licensing boards for regulars, homeopaths and eclectics, and the present single licensing board in which all "pathies" are finally represented. When the final record will be made, said Dr. Potter, the name of Dr. Henry Reed Hopkins will be found well near the topmost scroll on the roll of fame.

Dr. William C. Phelps, an ex-president of the society, and a fellow student with Dr. Hopkins, related some of the incidents of student days and early practice. The only time he had known the student Hopkins to fail was at the final examination for his degree, when his fellow students were surprised at his absence. Later, it was learned that, through no fault of his, he had not been born early enough to entitle him to admission. Dr. Phelps dwelt more on the humorous side of his friend's career.

Dr. Edward Clark, another ex-president of the society, and a former Health Officer of the City of Buffalo, briefly traced his acquaintance with Dr. Hopkins from the time when he first saw him as the chief censor whose endorsement was necessary for permission to enter the study of medicine, to the time when he assisted in fighting medical battles at home and at Albany.

Dr. DeLancey Rochester, in the course of very complimentary remarks, made one of the most important points, when he declared that a single act in Dr. Hopkins's career entitles him to immortality.

For years, the courts held different opinions as to what constitutes the practice of medicine. The celebrated Daniels's decision stood in the way of any material progress. Dr. Hopkins wrote the definition which clearly and concisely defines the practice of medicine, and this definition is incorporated in the medical law of 1907, thus depriving individual judges from making their own definitions.

Dr. P. W. Van Peyma, secretary of the Erie County Board of Midwifery Examiners, spoke of Dr. Hopkins's instrumentality in this direction in determining the qualification of midwives before being allowed to practise.

United States District Attorney John Lord O'Brian, paid tribute to the work of Dr. Hopkins in this community and also in the state at large. As attorney for the Erie County Medical Society, Mr. O'Brian had occasion to become intimately acquainted with Dr. Hopkins who, as Chairman of the Board of Censors, was never without a case for the attorney. Daily visits, or consultations by telephone were the rule. Nineteen abortionists were driven out of business at one time by the issuance of a government fraud order forbidding the use of mails.

Several abortionists were convicted, sentenced to imprisonment, and their license to practise cancelled. Other fakirs were imprisoned or driven away. In spite of the Daniels's precedent, Dr. Hopkins gained his point. When Mr. O'Brian went to Albany, as a member of Assembly, he found that Dr. Hopkins was the best known and most feared physician to the State Legislature.

President Wall called Vice-president Cohen to the chair and, after making a few appropriate remarks, presented to Dr. Hopkins, on behalf of the society, a beautiful silver loving cup containing the following inscription:

Henry Reed Hopkins, M. D.

1869—1909

From

THE MEDICAL SOCIETY OF THE COUNTY OF ERIE

*A token of their respect and esteem and of his being made an honorary member.*

In commemoration of forty years' service as a member of the Board of Censors.

Dr. Hopkins, in accepting the beautiful gift of the society, was visibly affected and, after briefly thanking the members, he addressed them as follows:

*Mr. President and Fellows:* Possibly you will pardon me if, in my attempted response to your kind utterances of this to me, ever memorable evening, there should appear a note of personality. You may, at least, depend upon my best efforts to keep the same from undue prominence.

However, when I remember the many nice things that you —Mr. President—and your several speakers have said, and recall my many failures and shortcomings concerning which you all have been most considerately silent, the provocation is distinctly in the direction not of an official response but rather of an appreciation, and that from a full heart.

I had the good fortune to have begun my medical education in 1865, near the middle of the century, and my medical fathers and teachers,—Pratt, Winnie, Moore, Hadley, Lee, White and Rochester,—were born near its beginning, so that from my teachers and from personal observation, I have some knowledge of the trend of the medical thought of the nineteenth century. It is not beneath the dignity of this occasion for me to remind you that the object, the trend of the medical thought of the twentieth century is quite different from that of the nineteenth century. That the paramount theme of the nineteenth century was the consideration of the individual, the diagnosis of his disease, the treatment of that disease, for the money there was in it.

The treatment of consumption, of scarlet fever, of typhoid fever by the allopathic system, by the homeopathic system, or by the eclectic system, these with their innumerable varieties and variations were the themes of discussion and medical conversation. The direction of this was to the individual to cure him of his illness for money. On the other hand, the medical orientation of the twentieth century is not the individual, not even the family, but is the State,—that the State may keep its individuals well, may prevent suffering, loss of time, and death, from the most frequent and the most deadly of all the diseases,—the preventable diseases.

In the nineteenth century, we had diagnosis for treatment. In the twentieth century, we have diagnosis for prevention. This thought of the twentieth century may, at first, seem to be like turning from the west to the east, but in fact it is but the logical and natural growth of the study of the diseases of the individual, the family, the town, the state.

This medical millenium of the twentieth century, the supreme vindication of the medical profession, when consumption, pneumonia, scarlet fever, diphtheria, typhoid fever and the other preventable diseases, will be as rare as are now rabies, smallpox and the plague, will come before the century is half over,—come within the lives of many of you who have the good fortune to work as long as I have worked.

The light of this medical millenium is already seen at Albany, and is guiding the policy of the State in medical legislation, as shown by the fact of State License for the practice of medicine, a revolution of enormous potentiality. State license was not born without pains and labor, and at that labor, the profession of Buffalo contributed a liberal proportion. From State license by three boards to State license by one board was again an instance of progress, not reorientation, and was in entire harmony with the medical evolution from the ideals of the nineteenth century to the vastly higher ideals of the twentieth century.

Those of you who have not given this subject much thought may consider what I have just said optimistic, beyond warrant and quite unreliable as a scientific statement, and I, therefore, beg such of you to calm your minds with the reflection that insight does give foresight; that God so made the world that foreknowledge is not only possible, but that foreknowledge may have scientific precision and accuracy. The man wise in mathematical principles does study the starry heavens, does learn the names of the different planets, does know their several sizes and weights, their several axial and orbital motions, their several distances from each other and from their common center, the sun, and can foretell their relative positions and eclipses years and centuries in the future, and that, to the day, the hour and the minute. By the similar operation of the mental faculties of observation and inference, the man learned in surgical principles does know something of the anatomy of the human body, something of the physico-chemistry of the different tissues and fluids of that body,—something of the life histories of some of the enemies of that body,—and from this knowledge he can and does foreknow and foretell that when necessary he can with safety open the cavity of the knee, of the peritoneum, of the pleura, of the pericardium and of the pia mater.

In like manner, the hygienist, making use of those same priceless mental faculties,—observation and inference,—attains a clear vision of the achievements of preventive medicine of the future, and in that future, which many of you will live to enjoy, there will be no prominence in the list of causes of death of consumption, pneumonia, scarlet fever, diphtheria, typhoid fever or the other communicable diseases; that, just as typhus fever, the plague and smallpox—diseases which, for centuries, caused a majority of all the deaths,—are now, to most countries, mere matters of ancient history; so the historian of the closing years of this century will recount the interesting facts as to the disappearance of the chief causes of our present death-rate.

I have said that the insight of the hygienist makes this expectation legitimate and natural; and have only to add that this medical millenium, while it is the pride, is not the boast or the work of the hygienist, or any other specialist in medicine or in science. When this consummation, devoutly to be wished, comes to bless the earth, it will be seen to be the resultant of many discoveries as to the cause of disease, the perfection of many methods as to the prevention of disease, but above all other forces will rate the work of the little red schoolhouse on the hillside where our humble millions are taught those priceless lessons of the three R's (reading, riting and rithmetic) where intelligent public opinion is made possible, because medical convictions expressed and crystallised in health laws and health ordinances, only become effective when loyally supported and executed by intelligent public opinion.

That the State of New York may, in due time, enjoy this blessing, there must be in the state one medical profession, compact and effective in unity of organisation, of such preparedness as is only possible when its individual members are men of high ideals, inspired with the hope and expectation of great accomplishments in the field of preventive medicine.

I have the belief, nay more, the conviction, that aside from any complimentary or personal element in the proceedings of this meeting, this review of the official activities of your recent representatives can but act profitably, to widen our ideas of the purposes, the scope, the responsibility of our medical societies, to stimulate pride in our profession and its high and ennobling purposes. Should something like this be the result of our evening, you, Mr. President and speakers, will not have striven or spoken in vain.

At the conclusion of Dr. Hopkins's remarks, Dr. Stockton moved that Dr. Hopkins's address be embodied in full in the minutes of this meeting. Motion was adopted.

President Wall then declared the meeting adjourned, and an impromptu reception to Dr. Hopkins immediately followed, when personal congratulations were tendered by the many physicians present.

A collation was served and, for a considerable period of time, the members remained in social intercourse.

